# Circulating Fibroblast Growth Factor-23 Levels are Associated with an Increased Risk of Anemia Development in Patients with Nondialysis Chronic Kidney Disease

**DOI:** 10.1038/s41598-018-25439-z

**Published:** 2018-05-08

**Authors:** Ki Heon Nam, Hyoungnae Kim, Seong Yeong An, Misol Lee, Min-Uk Cha, Jung Tak Park, Tae-Hyun Yoo, Kyu-Beck Lee, Yeong-Hoon Kim, Su-Ah Sung, Joongyub Lee, Shin-Wook Kang, Kyu Hun Choi, Curie Ahn, Seung Hyeok Han

**Affiliations:** 1 Department of Internal Medicine, College of Medicine, Institute of Kidney Disease Research, Yonsei University, Seoul, Korea; 2 Department of Internal Medicine, Sungkyunkwan University School of Medicine, Kangbuk Samsung Hospital, Seoul, Korea; 3 Department of Internal Medicine, Busan Paik Hospital, College of Medicine, Inje University, Busan, Korea; 4 Department of Internal Medicine, Eulji General Hospital, Eulji School of Medicine, Seoul, Korea; 5 Medical Research Collaborating Center, Seoul National University Hospital and Seoul National University College of Medicine, Seoul, Korea; 6 Department of Internal Medicine, Seoul National University College of Medicine, Seoul, Korea; 7 Department of Internal Medicine, College of Medicine, Severance Biomedical Science Institute, Brain Korea 21 PLUS, Yonsei University, Seoul, Korea

## Abstract

Fibroblast growth factor-23 (FGF23) is an established biomarker of adverse outcomes in patients with chronic kidney disease (CKD). Several cross-sectional studies have suggested a possible association between FGF23 and anemia in these patients. In this large-scale prospective cohort study, we investigated this relationship and examined whether high FGF23 levels increase the risk of incident anemia. This prospective longitudinal study included 2,089 patients from the KoreaN cohort study for Outcome in patients With CKD. Anemia was defined as hemoglobin level <13.0 g/dl (men) and <12.0 g/dl (women). Log-transformed FGF23 significantly correlated with hepcidin but inversely correlated with iron profiles and hemoglobin. Multivariate logistic regression showed that log-transformed FGF23 was independently associated with anemia (odds ratio [OR], 1.14; 95% confidence interval [CI], 1.04–1.24, *P* = 0.01). Among 1,164 patients without anemia at baseline, 295 (25.3%) developed anemia during a median follow-up of 21 months. In fully adjusted multivariable Cox models, the risk of anemia development was significantly higher in the third (hazard ratio [HR], 1.66; 95% CI, 1.11–2.47; *P* = 0.01) and fourth (HR, 1.84; 95% CI, 1.23–2.76; *P* = 0.001) than in the first FGF23 quartile. In conclusion, high serum FGF23 levels were associated with an increased risk for anemia in patients with nondialysis CKD.

## Introduction

Anemia is one of the most common complications in chronic kidney disease (CKD), and its prevalence increases progressively as kidney function declines^1^. The recent National Health and Nutrition Examination Survey reported that anemia was twice as prevalent in persons with CKD (15.4%) as that in the general population (7.6%) in the United States^2^. In addition, anemia is closely associated with poor quality of life and adverse outcomes such as left ventricular hypertrophy, cardiovascular events, and increased mortality in patients with CKD^3–5^. Although the mechanism for anemia in CKD is primarily due to failure of erythropoietin (EPO) production in response to decreased hemoglobin concentration^6^, other potential factors such as chronic inflammation, iron deficiency, malnutrition, increased destruction of red blood cells, and vitamin D deficiency also contribute to the pathogenesis of renal anemia^7–9^.

Fibroblast growth factor-23 (FGF23) is a bone-derived hormone that is crucial for maintaining normal phosphate and vitamin D homeostasis^10,11^. FGF23 levels increase gradually with diminishing renal function, and it seems to be an adaptive mechanism to prevent hyperphosphatemia^12^. Recently, elevated FGF23 levels have been shown to be associated with adverse outcomes, such as progression of kidney disease^13,14^, vascular calcification^15^, left ventricular hypertrophy^16^, cardiovascular events^17,18^, and increased mortality^14,17,19^. Considering that FGF23 and anemia are nontraditional risk factors for adverse outcomes in patients with CKD, and both are reciprocally changed according to kidney function, it would be intriguing to explore the relationship between FGF23 and anemia in CKD. Interestingly, the inhibitory effect of FGF23 on erythropoiesis was demonstrated in an experimental study^20^. However, only few human studies have suggested a possible association between FGF23 and anemia in patients with CKD^21–23^. In most studies, the causality between FGF23 and anemia could not be explained because this relationship was analyzed through a cross-sectional approach. The purpose of this study was, therefore, to further clarify the relationship between FGF23 levels and anemia in patients with CKD and to examine whether high FGF23 levels increase the future development of anemia, in a large-scale prospective cohort study.

## Materials and Methods

### Study design and population

**Figure 1:**
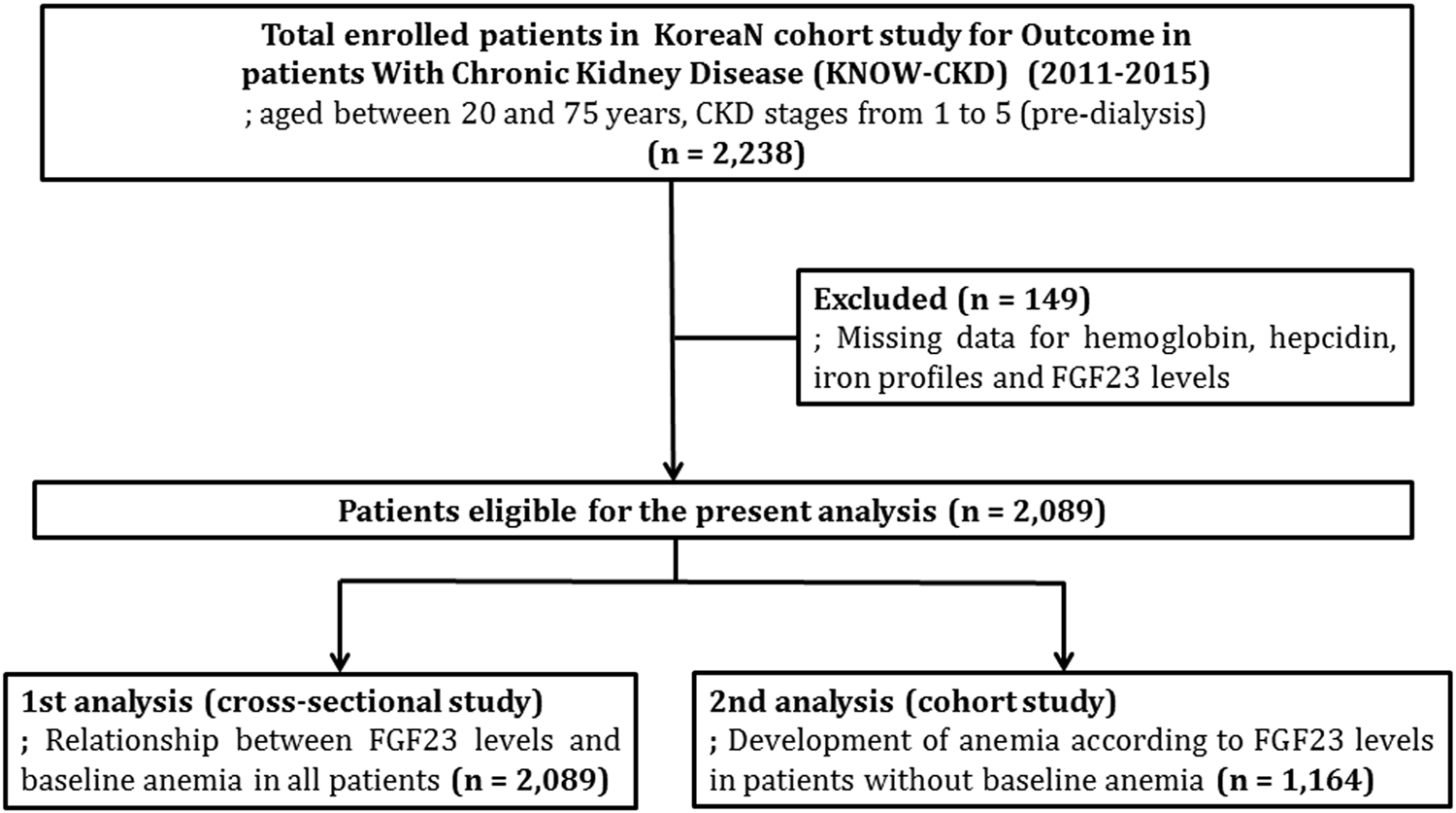
Flow chart for patients enrollment and analyses.

The KoreaN cohort study for Outcome in patients With Chronic Kidney Disease (KNOW-CKD) is a prospective nationwide cohort study investigating various clinical courses and risk factors for the progression of CKD in Korean patients. Patients aged between 20 and 75 years with CKD stage 1–5 before dialysis who voluntarily provided informed consent were enrolled from nine university-affiliated tertiary-care hospitals throughout Korea between June 2011 and February 2015. The detailed design and method of the study have previously been described elsewhere (NCT01630486 at http://www.clinicaltrials.gov)^24^. Among 2,238 patients in the KNOW-CKD cohort, 149 patients with missing data for hemoglobin, hepcidin, iron profiles, and C-terminal FGF23 (FGF23) levels were excluded. Finally, 2,089 patients were included in the present analysis (Fig. 1).

### Data collection

Baseline sociodemographic information and laboratory data were obtained from the KNOW-CKD database. The resting blood pressure (BP) in the clinic was measured with an electronic sphygmomanometer, and body mass index (BMI) was determined using the formula “weight (kg)/height (m^2^). Serum sample collected for initial study measurements sent to the central laboratory of the KNOW-CKD study (Lab Genomics, Seongnam, Republic of Korea) and were stored at −80 °C in deep freezer. Along with blood samples, urine samples were also immediately sent to central lab and subject for proteinuria measurement. Laboratory tests were obtained every 6 months in the first year and then annually thereafter. Serum creatinine was measured using an isotope dilution mass spectrometry-traceable method, and estimated glomerular filtration rate (eGFR) was calculated using the four-variable Modification of Diet in Renal Disease equation^25^. Serum levels of hepcidin were measured using commercially available ELISA kits (DRG Instruments GmBH, Marburg, Germany). Anemia was defined as hemoglobin levels of <13.0 g/dL for men and <12.0 g/dL for women according to World Health Organization (WHO) criteria. Transferrin saturation (TSAT) was calculated using the ratio of serum iron and total iron-binding capacity. Iron deficiency was defined as ferritin <100 ng/mL or TSAT <20%.

Serum C-terminal FGF23 concentration was measured using enzyme-linked immunosorbent assay (ELISA; Immutopics, San Clemente, CA, USA). Regarding sensitivity of FGF23 assay, the 95% confidence limit on 20 duplicate determinations of the 0 RU/ml standard is 1.5 RU/ml. Precision of FGF23 assay was carried out for quality control measures. Intra-assay precision was calculated from 20 duplicate determinations of two samples each performed in a single assay. Coefficient of variations for FGF level of 33.7 and 302 RU/ml were 2.4% and 1.4%, respectively. In addition, inter-assay precision was calculated from duplicate determinations of two samples performed in 10 assays. Coefficient of variations for FGF level of 33.6 and 293 RU/mL were 4.7% and 2.4%, respectively.

### Study endpoint

We first performed a cross-sectional analysis to clarify the relationship between serum FGF23 levels and anemia in 2,089 patients using the baseline data. Then, we further examined whether elevated FGF23 levels increase the future development of anemia in 1,164 patients who had no anemia at baseline (Fig. 1). For this analysis, the primary outcome was newly developed anemia during the follow-up period.

### Statistical analyses

All analyses were performed with IBM SPSS Statistics version 21 (IBM Corp., Armonk, NY, USA) and SAS version 9.4 (SAS Institute, Cary, NC, USA). All variables with a normal distribution were expressed as mean ± standard deviation. If data did not have a normal distribution, they were expressed as median and interquartile range (IQR). Categorical variables were expressed as number and proportion. Comparisons were made using one-way analysis of variance for continuous variables and the chi-square test for categorical variables, as required. Pearson’s correlation test was used to evaluate the relationship between covariables, and a multivariable linear regression analysis for hemoglobin level was performed after adjustment for age, sex, presence of diabetes mellitus (DM), BMI, systolic BP (SBP), Charlson comorbidity index (CCI), smoking status, eGFR, albumin, phosphorus, 1,25(OH)2 vitamin D, presence of iron deficiency, hepcidin, C-reactive protein (CRP), proteinuria, and anemia treatment including iron replacement and EPO-stimulating agent (ESA). In addition, a multivariable-adjusted logistic regression analysis was conducted to determine whether FGF23 was associated with anemia as defined based on WHO criteria. The results were presented as odds ratios (ORs) and 95% confidence intervals (CIs). Among 1,164 patients without baseline anemia, the cumulative anemia-free survival rates were estimated using the Kaplan–Meier method and differences between survival curves were compared with the log-rank test. Furthermore, multivariate Cox regression models for the development of anemia were constructed after rigorous and stepwise adjustments for confounding factors. Model 1 was the unadjusted model with no covariables. Model 2 included adjustment for age, sex, DM, BMI, SBP, CCI, and smoking. We constructed model 3 by adding eGFR, albumin, phosphorus, 1,25(OH)2 vitamin D, presence of iron deficiency, hepcidin, CRP, and proteinuria to model 2. Moreover, model 4 included iron replacement and ESA therapy in addition to model 3 variables. The results were presented as hazard ratios (HRs) and 95% CIs. We also examined the association between FGF23 levels and the development of anemia through subgroup analysis using a fully adjusted multivariate Cox regression model. The patients were stratified according to sex, history of DM, presence of iron deficiency, treatment with renin-angiotensin system (RAS) blockers, and median values of age, SBP, BMI, CCI, eGFR, albumin, CRP, and 1,25(OH)2 vitamin D. *P* < 0.05 was considered statistically significant for all analyses.

### Ethics statement

We carried out the study in accordance with the Declaration of Helsinki, and the study was approved by the institutional review board of each participating clinical center, as follows: Seoul National University Hospital (1104–089–359), Seoul National University Bundang Hospital (B-1106/129-008), Yonsei University Severance Hospital (4-2011-0163), Kangbuk Samsung Medical Center (2011-01-076), Seoul St. Mary’s Hospital (KC11OIMI0441), Gil Hospital (GIRBA2553), Eulji General Hospital (201105-01), Chonnam National University Hospital (CNUH-2011-092), and Pusan Paik Hospital (11-091) in 2011.

## Results

### Patient characteristics according to fibroblast growth factor-23

The median serum FGF23 level was 19.6 RU/ml. The median value of FGF23 in each quartiles, from the first to the fourth, was 0.0 (IQR, 0.0–0.4; range, 0.0–1.7), 9.5 (IQR, 4.8–15.9; range, 1.8–19.6), 25.5 (IQR, 22.3–29.8; range, 19.7–34.6), and 52.9 (IQR, 41.3–75.3; range, 34.7–774.0). According to FGF23 quartiles, we categorized the study subjects into four groups and compared their baseline characteristics (Table 1). The mean age was 53.6 ± 12.2 years and 1,275 patients (61.0%) were male. The mean eGFR was 50.3 ± 30.2 mL·min^−1^·1.73 m^−2^ and was significantly lower in high FGF23 quartiles than in low quartiles (*P* < 0.001). The prevalence of DM and serum levels of hepcidin, phosphorus, and intact parathyroid hormone were higher, whereas calcium and 1,25(OH)2 vitamin D levels were lower in high FGF23 quartiles (*P* < 0.001 for all). The mean hemoglobin levels were 12.8 ± 2.0 g/dL and were significantly lower in high FGF23 quartiles (*P* < 0.001). Regarding iron profiles, serum iron levels and TSAT were also lower in high FGF23 quartiles. However, serum ferritin levels did not differ among quartiles. There were more patients who received iron replacement and ESA therapy in the high FGF23 quartiles.

**Table 1:** Baseline characteristics of patients according to FGF23 quartile.

Variables	FGF23 quartiles				Total	p-value
	Quartile 1 (0.0–1.7)	Quartile 2 (1.8–19.6)	Quartile 3 (19.7–34.6)	Quartile 4 (34.7–774.0)		
Number	522	522	523	522	2,089	
Age (years)	52.6 ± 12.4	53.0 ± 12.2	54.6 ± 11.9	54.0 ± 12.4	53.6 ± 12.2	0.04
Sex (Male, %)	321 (61.5)	318 (60.9)	345 (66.0)	291 (55.7)	1,275 (61.0)	0.01
Smoking (n, %)	238 (45.6)	232 (44.8)	262 (50.1)	240 (46.0)	972 (46.5)	0.41
DM (n, %)	140 (26.8)	145 (27.8)	189 (36.1)	236 (45.2)	710 (34.0)	<0.001
HTN (n, %)	491 (94.1)	503 (96.4)	508 (97.1)	506 (96.9)	2,008 (96.9)	0.41
BMI (kg/m^2^)	24.5 ± 3.2	24.6 ± 3.4	24.7 ± 3.5	24.4 ± 3.5	24.5 ± 3.4	0.65
SBP (mmHg)	127.9 ± 15.7	126.6 ± 14.7	127.7 ± 16.5	132.0 ± 18.3	128.5 ± 16.5	<0.001
DBP (mmHg)	77.1 ± 11.3	76.2 ± 10.8	76.1 ± 10.9	78.0 ± 11.6	94.1 ± 11.7	0.02
MAP (mmHg)	94.0 ± 11.5	93.0 ± 11.0	93.3 ± 11.5	96.0 ± 12.6	76.9 ± 11.2	<0.001
Anemia (n, %)	165 (31.6)	178 (34.1)	253 (48.4)	329 (63.0)	925 (44.3)	<0.001
Charlson comorbidity index	1.9 ± 1.6	2.1 ± 1.6	2.5 ± 1.5	2.8 ± 1.6	2.3 ± 1.6	<0.001
Creatinine (mg/dl)	1.5 ± 0.8	1.5 ± 0.8	1.9 ± 1.1	2.3 ± 1.5	1.8 ± 1.1	<0.001
eGFR (ml/min/1.73 m^2^)	59.1 ± 30.1	56.8 ± 31.2	45.6 ± 26.7	39.8 ± 28.5	50.3 ± 30.2	<0.001
CKD stage						
1 (n, %)	116 (22.2)	106 (20.3)	61 (11.7)	53 (10.2)	336 (16.1)	<0.001
2 (n, %)	137 (26.2)	105 (20.1)	86 (16.4)	64 (12.3)	392 (18.8)	<0.001
3 (n, %)	183 (35.1)	218 (41.8)	211 (40.3)	170 (32.6)	782 (37.4)	0.006
4 (n, %)	72 (13.8)	82 (15.7)	133 (25.4)	162 (31.0)	439 (21.0)	<0.001
5 (n, %)	14 (2.7)	11 (2.1)	32 (6.1)	73 (14.0)	130 (6.2)	<0.001
WBC (x10^3^/mm^3^)	6.5 ± 2.0	6.6 ± 1.8	6.7 ± 1.9	6.6 ± 2.0	6.6 ± 1.9	0.26
Hemoglobin (g/dl)	13.3 ± 1.8	13.2 ± 1.9	12.8 ± 2.1	12.0 ± 2.0	12.8 ± 2.0	<0.001
Platelet (x10^3^/mm^3^)	228.8 ± 62.2	231.0 ± 58.0	231.9 ± 59.8	232.2 ± 65.7	231.0 ± 61.5	0.81
Iron (µg/dl)	95.5 ± 35.0	98.1 ± 36.6	93.6 ± 34.6	83.2 ± 32.8	92.6 ± 35.2	<0.001
Ferritin (ng/ml)*	101.5 (58.6–176.0)	91.0 (52.2–161.9)	100.3 (53.6–186.3)	101.0 (49.4–175.2)	98.3 (53.3–175.7)	0.45
Transferrin saturation (%)	32.2 ± 11.7	33.1 ± 12.7	31.8 ± 11.8	29.4 ± 11.9	31.7 ± 12.1	<0.001
Hepcidin (ng/ml)*	11.5 (5.8–22.8)	11.7 (6.1–21.5)	14.4 (7.2–25.8)	16.5 (7.8–30.4)	13.4 (6.6–25.1)	<0.001
Total cholesterol (mg/dl)	176.5 ± 38.3	175.4 ± 37.3	172.8 ± 39.7	173.1 ± 42.2	174.4 ± 39.4	0.37
Albumin (g/dl)	4.2 ± 0.4	4.2 ± 0.4	4.2 ± 0.4	4.1 ± 0.5	4.2 ± 0.4	<0.001
Calcium (mg/dl)	9.2 ± 0.5	9.2 ± 0.5	9.1 ± 0.5	9.0 ± 0.6	9.1 ± 0.5	<0.001
Phosphate (mg/dl)	3.6 ± 0.6	3.6 ± 0.6	3.7 ± 0.6	4.0 ± 0.8	3.7 ± 0.7	<0.001
iPTH (pg/ml)*	44.5 (29.0–68.9)	46.1 (31.2–70.3)	53.0 (35.0–86.3)	69.0 (41.6–121.8)	51.6 (33.2–84.7)	<0.001
1,25(OH)2 vitamin D (pg/ml)	32.1 ± 17.2	29.2 ± 13.3	27.4 ± 12.7	25.7 ± 5.3	28.6 ± 14.4	<0.001
FGF23 (RU/ml)*	0.03 (0.0–0.4)	9.5 (4.8–15.9)	25.5 (22.3–29.8)	52.9 (41.3–75.3)	19.6 (1.7–34.6)	<0.001
CRP (mg/dl)*	0.7 (0.2–1.6)	0.6 (0.2–1.4)	0.6 (0.3–1.6)	0.7 (0.2–2.2)	0.6 (0.2–1.6)	0.04
Proteinuria (g/24 h)*	0.4 (0.1–1.1)	0.5 (0.2–1.2)	0.7 (0.2–1.9)	0.7 (0.3–2.2)	0.5 (0.2–1.6)	<0.001
Treatment						
RAS blockers (n, %)	446 (85.4)	452 (86.6)	436 (83.4)	448 (85.8)	1,782 (85.3)	0.50
Statin (n, %)	262 (50.2)	273 (52.3)	277 (53.0)	267 (51.1)	1,079 (51.8)	0.84
Iron replacement (n, %)	50 (9.6)	62 (11.9)	82 (15.7)	113 (21.6)	179 (8.6)	<0.001
ESA therapy (n, %)	19 (3.6)	27 (5.2)	35 (6.7)	78 (14.9)	167 (7.5)	<0.001

All data are expressed as mean ± SD or *median (and interquartile range)

Abbreviations: FGF23, fibroblast growth factor 23; DM, diabetes mellitus; HTN, hypertension; BMI, body mass index; SBP, systolic blood pressure; DBP, diastolic blood pressure; MAP, mean arterial pressure; eGFR, estimated glomerular filtration rate; CKD, chronic kidney disease; WBC, white blood cell; iPTH, intact parathyroid hormone; S-Klotho, soluble α-Klotho; CRP, C-reactive protein; RAS, renin-angiotensin system; ESA, erythropoiesis-stimulating agent.

### Relationship between fibroblast growth factor-23 levels and baseline anemia

**Figure 2:**
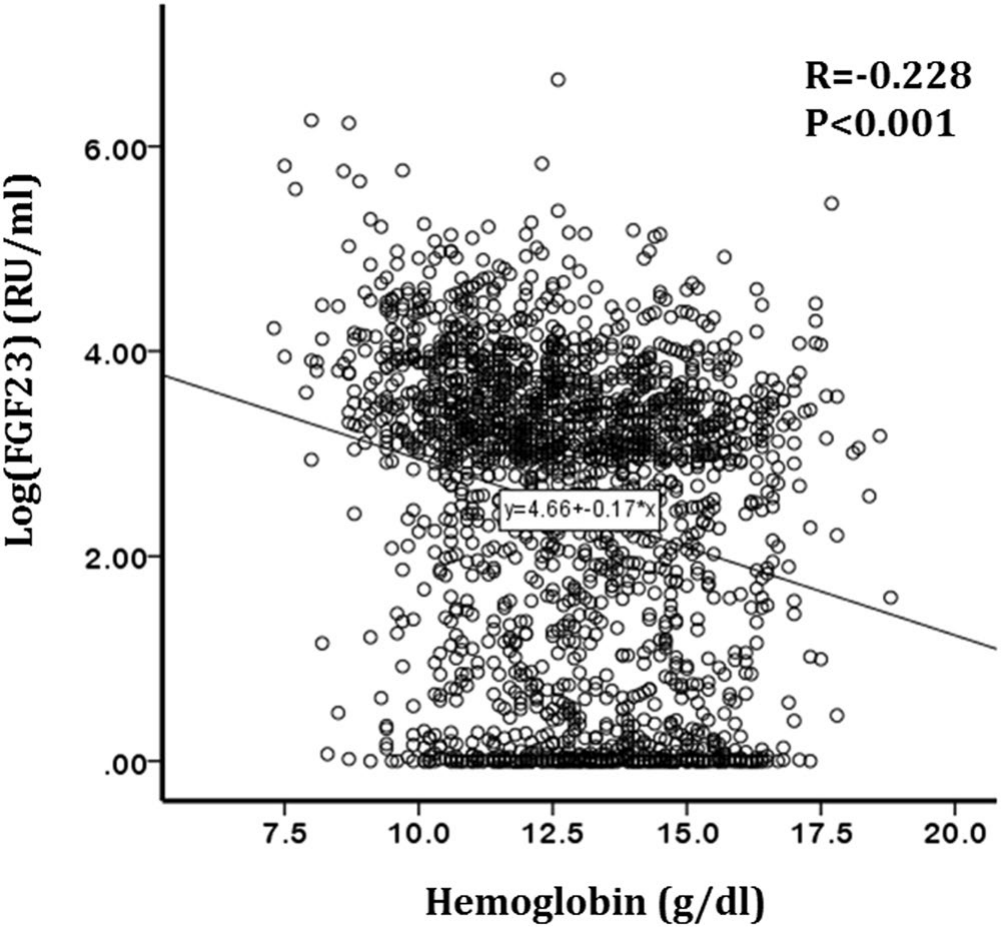
Correlation between FGF23 and hemoglobin.

In Pearson correlation analyses, log-transformed FGF23 was inversely correlated with eGFR, albumin, calcium, 1,25(OH)2 vitamin D, iron, TSAT, and hemoglobin (Fig. 2), whereas it was positively correlated with CCI, phosphorus, intact parathyroid hormone, hepcidin, and proteinuria. However, age, BMI, CRP, and ferritin were not correlated with FGF23. We then performed in-depth analyses to clarify the association between FGF23 and anemia. In a multivariable linear regression analysis adjusted for confounding factors, there was a significant inverse relationship between log-transformed FGF23 and hemoglobin levels (β = −0.067, *P* = 0.004; Table 2). This finding was further strengthened in a multivariable logistic model (Table 3). After rigorous adjustment of confounders, log-transformed FGF23 was independently associated with anemia (OR, 1.14; 95% CI, 1.04–1.24, *P* = 0.01). When FGF23 quartiles were entered as a categorical variable in the model, the highest quartile of FGF23 was significantly associated with anemia compared with the lowest quartile (OR, 1.72; 95% CI, 1.19–2.50, *P = *0.004).

**Table 2:** Multivariable linear regression analysis of factors associated with hemoglobin levels (cross- sectional analysis).

Variables	Hemoglobin (g/dl)				
	β	95% CI			*P-*value
		Lower	Upper		
Age (per 10 years)	−0.032	−0.094		0.030	0.32
Sex (male)	1.296	1.098		1.494	<0.001
BMI (per 1 kg/m^2^)	0.079	0.058		0.100	<0.001
Diabetes mellitus	−0.366	−0.585		−0.147	0.001
Smoking	−0.065	−0.245		0.115	0.48
CCI (per 1score)	−0.100	−0.179		−0.021	0.01
SBP (per 1 mmHg)	−0.003	−0.007		0.002	0.21
eGFR (per 1 ml/min per 1.73 m^2^)	0.015	0.011		0.018	<0.001
Albumin (per 1 g/dl)	0.926	0.732		1.119	<0.001
Phosphorus (per 1 mg/dl)	−0.453	−0.568		−0.338	<0.001
1,25(OH)_2_ vitamin D (per 1 pg/ml)	0.001	−0.004		0.006	0.66
Iron deficiency	−0.325	−0.484		−0.166	<0.001
Hepcidin (per 1 log)*	−0.150	−0.234		−0.067	<0.001
CRP (per 1 log)*	−0.019	−0.122		0.085	0.72
FGF23 (per 1 log)*	−0.067	−0.114		−0.021	0.004
Proteinuria (per 1 log)*	0.044	−0.006		0.094	0.09
Iron replacement	−0.862	−1.079		−0.645	<0.001
ESA therapy	−0.361	−0.653		−0.069	0.02

Abbreviations: BMI, body mass index; CCI, Charlson comorbidity index; SBP, systolic blood pressure; eGFR, estimated glomerular filtration rate; CRP, C-reactive protein; FGF23, fibroblast growth factor 23; ESA, erythropoiesis-stimulating agent. *log transformed.

**Table 3:** Multiple logistic regression analysis of factors associated with anemia (cross- sectional analysis).

	Univariate analysis		Multivariate analysis*	
	OR (95% CI)	P-value	OR (95% CI)	P-value
FGF23 quartile				
Quartile 1 (n = 522)	Reference		Reference	
Quartile 2 (n = 522)	1.11 (0.86–1.44)	0.43	0.97 (0.67–1.40)	0.87
Quartile 3 (n = 523)	2.10 (1.63–2.70)	<0.001	1.31 (0.91–1.88)	0.14
Quartile 4 (n = 522)	3.49 (2.70–4.50)	<0.001	1.72 (1.19–2.50)	0.004
FGF23 (per 1 log)	1.36 (1.26–1.45)	<0.001	1.14 (1.04–1.24)	0.01

Note: FGF23 entered as a categorical and continuous form (log transformed).

*Adjusted for age, sex, DM, BMI, SBP, CCI, smoking, eGFR, albumin, phosphorus, 1,25(OH)_2_ vitamin D, iron deficiency, log-transformed hepcidin, log-transformed CRP, log-transformed proteinuria, and iron/ESA therapy.

Abbreviations: FGF23, fibroblast growth factor 23; DM, diabetes mellitus; BMI, body mass index; SBP, systolic blood pressure; CCI, Charlson comorbidity index; eGFR, estimated glomerular filtration rate; CRP, C-reactive protein; ESA, erythropoiesis-stimulating agent.

### High fibroblast growth factor-23 levels increase the development of anemia

**Figure 3:**
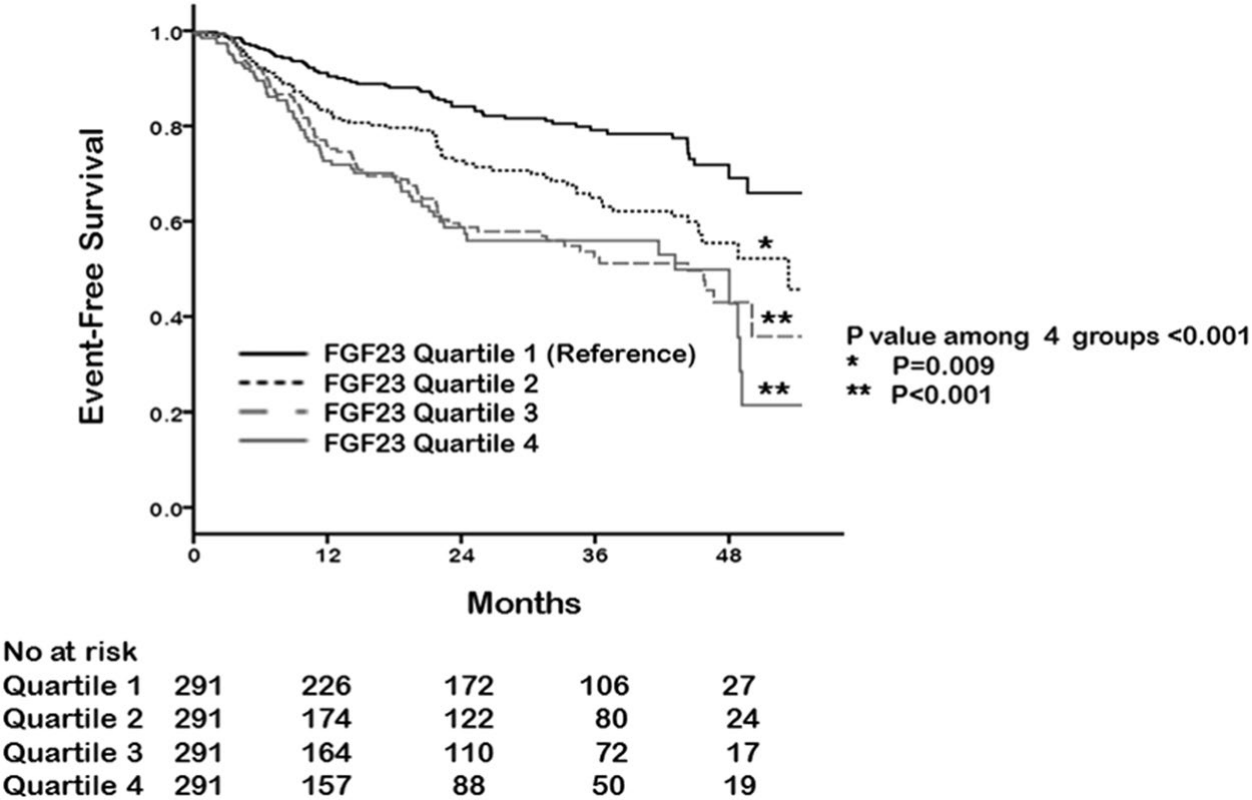
Cumulative curves of anemia development according to FGF23 quartiles for patients without anemia at baseline.

We further investigated whether FGF23 levels increase the future development of anemia. To this end, we selected 1,164 patients without anemia at baseline measurement. During the median follow-up duration of 21 (IQR, 7–38) months, 295 (25.3%) patients developed anemia. Anemia occurred in 48 (16.5%), 63 (21.6%), 91 (31.3%), and 93 (32.0%) patients in the first, second, third, and fourth quartile of FGF23, respectively (*P* < 0.001, Table 4). The Kaplan–Meier curves for anemia-free survival according to FGF23 quartiles are presented in Fig. 3. The time to the development of anemia was significantly shorter in high FGF23 quartiles (*P* = 0.009 for first vs. second; *P* < 0.001 for first vs. third and fourth). An in-depth analysis on the association between FGF23 levels and the development of anemia was performed using multivariable Cox regression models (Table 5). The crude HRs for incident anemia were 1.65 (95% CI, 1.13–2.40; *P* = 0.009), 2.55 (95% CI, 1.80–3.62; *P* < 0.001), and 2.94 (95% CI, 2.07–4.17; *P* < 0.001) in the second, third, and fourth quartile compared with the first quartile. Further stepwise multivariable adjusted models yielded similar results. In the fully adjusted model, the risk of developing anemia was significantly higher in the third (HR, 1.66; 95% CI, 1.11–2.47; *P* = 0.01) and fourth (HR, 1.84; 95% CI, 1.23–2.76; *P* = 0.003) quartile of FGF23 compared with the first quartile. When FGF23 was treated as a continuous variable, a significant association between FGF23 and anemia remained unaltered (HR for every 1 log increase, 1.17; 95% CI, 1.07–1.29; *P* = 0.001).

**Table 4:** Clinical outcomes according to baseline FGF23 quartile.

	All	Patients without baseline anemia (n = 1,164)				
		Quartile 1	Quartile 2	Quartile 3	Quartile 4	*p*-for trend
		N (%)	N (%)	N (%)	N (%)	
Newly developed anemia during follow-up	295 (25.3%)	48 (16.5%)	63 (21.6%)	91 (31.3%)	93 (32.0%)	<0.001

Abbreviations: FGF23, fibroblast growth factor 23.

**Table 5:** Multivariable Cox regression model for the development of anemia.

	Model 1^a^		Model 2^b^		Model 3^c^		Model 4^d^	
	HR (95% CI)	P-value	HR (95% CI)	P-value	HR (95% CI)	P-value	HR (95% CI)	P-value
FGF23 quartile								
Quartile 1	Reference		Reference		Reference		Reference	
Quartile 2	1.65 (1.13–2.40)	0.009	1.48 (1.02–2.16)	0.04	1.23 (0.81–1.87)	0.34	1.22 (0.80–1.85)	0.36
Quartile 3	2.55 (1.80–3.62)	<0.001	2.15 (1.51–3.07)	<0.001	1.71 (1.15–2.54)	0.008	1.66 (1.11–2.47)	0.01
Quartile 4	2.94 (2.07–4.17)	<0.001	2.42 (1.70–3.45)	<0.001	1.95 (1.31–2.90)	0.001	1.84 (1.23–2.76)	0.003
FGF23 (per 1 log)	1.32 (1.22–1.43)	<0.001	1.26 (1.16–1.36)	<0.001	1.19 (1.08–1.31)	<0.001	1.17 (1.07–1.29)	0.001

Note: FGF23 entered as a categorical and continuous form (log transformed).

^a^Unadjusted model.

^b^Adjusted for age, sex, DM, BMI, SBP, CCI, smoking status.

^c^Adjusted for Model 2 + eGFR, albumin, phosphorus, 1,25(OH)_2_ vitamin D, iron deficiency, hepcidin*, CRP*, and proteinuria*.

^d^Adjusted for Model 3 + iron replacement and ESA therapy.

*Variables were log transformed.

Abbreviations: FGF23, fibroblast growth factor 23; DM, diabetes mellitus; BMI, body mass index; SBP, systolic blood pressure; CCI, Charlson comorbidity index; eGFR, estimated glomerular filtration rate; CRP, C-reactive protein; ESA, erythropoiesis-stimulating agent.

### Subgroup analyses

**Figure 4:**
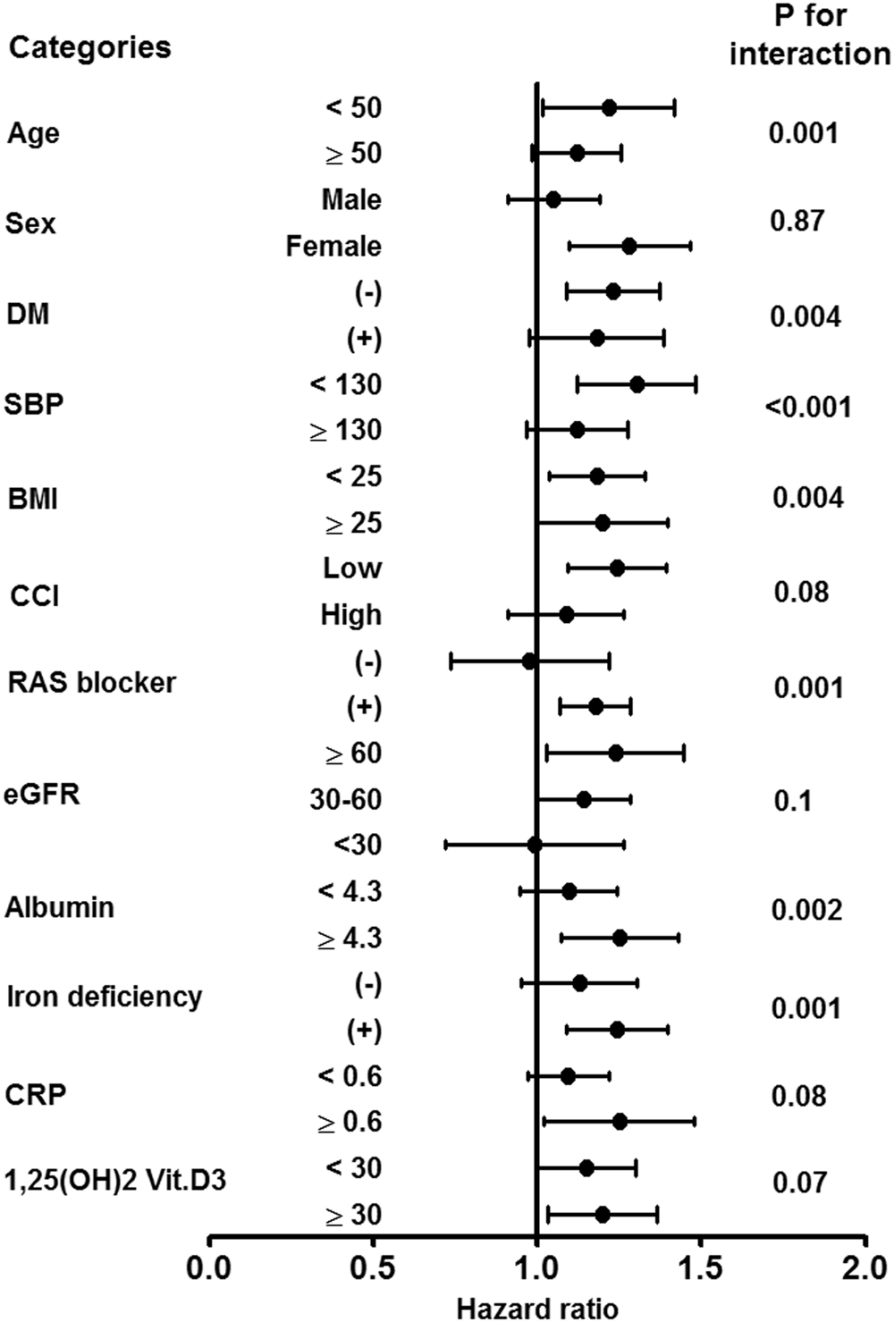
Subgroup analyses for association between FGF23 and anemia development. Abbreviations: DM, diabetes mellitus; SBP, systolic blood pressure; BMI, body mass index; CCI, Charlson comorbidity index; RAS, renin-angiotensin system; eGFR, estimated glomerular filtration rate; CRP, C-reactive protein.

We also investigated the relationship between FGF23 and the development of anemia in several subgroups stratified by age, sex, presence of diabetes, SBP, BMI, CCI, eGFR, albumin, 1,25(OH)2 vitamin D, CRP, and iron deficiency status. The results showed the consistent direction of the impact of FGF23 with respect to anemia in most stratified groups (Fig. 4). Notably, this association was observed particularly in nondiabetics, patients aged <50 years, patients treated with RAS blockers, patients with iron deficiency, or patients with SBP <130, BMI <25 kg/m^2^, CCI <3, eGFR >30 ml·min^−1^·1.73 m^−2^ or albumin ≥ 4.3 g/dl.

## Discussion

In this prospective cohort study, we demonstrated the inverse relationship between serum FGF23 and hemoglobin in patients with nondialysis CKD. In addition, we also showed that high serum FGF23 levels were significantly associated with an increased risk for the development of anemia even after a rigorous adjustment for multiple confounding factors. This association was particularly evident in patients treated with RAS blockers, patients with young age, relatively preserved eGFR, low comorbid burden, and iron deficiency. Our findings are of great clinical importance, as anemia is a frequent complication in patients with CKD and is associated with adverse outcomes^3–5^. Although there are well-established risk factors for anemia, our study suggests that FGF23 can also be a useful biomarker of incident anemia in patients with nondialysis CKD.

Studies that have evaluated the relationship between FGF23 and anemia have yielded conflicting results. To date, there have been only five studies that have specifically addressed this issue. In a study by Akalin *et al*., there was no association between FGF23 and hemoglobin levels in 89 patients undergoing hemodialysis^26^. In addition, a recent observational study by Honda *et al*. showed no significant relationship between FGF23 and hemoglobin levels in 282 patients undergoing hemodialysis^27^. These findings were contradicted by two other studies: one on patients undergoing peritoneal dialysis and one on patients with CKD before dialysis treatment^21,22^. These studies showed a significant inverse association between FGF23 and anemia. However, these studies are limited by their small sample size and cross-sectional analyses. Furthermore, as dialysis patients are more likely to receive iron preparation and ESA, interpretation should be made carefully in these patients. Of note, our study has a larger sample size than most previous studies, which thus assures power to detect statistical significance. In addition, we demonstrated that high FGF23 level increased the future development of anemia in a longitudinal observation of a CKD cohort. Our findings particularly corroborated findings from a recent publication by the Chronic Renal Insufficiency Cohort Study investigators^23^, indicating that elevated FGF23 is associated with prevalent anemia, change in hemoglobin over time, and the development of anemia.

The mechanism responsible for FGF23-associated anemia is unknown. Although anemia in CKD is a multifactorial disorder, it is well explained by insufficient EPO, a hormone that stimulates red blood cell production in the bone marrow in response to low oxygen levels in the blood^6,8^. EPO production is impaired at any given hematocrit concentration in patients with decreased renal function^6^. Interestingly, one experimental study found that loss of FGF23 resulted in markedly augmented erythropoiesis in peripheral blood and bone marrow of young adult mice, which can be accounted for by elevated serum EPO levels and EPO mRNA synthesis in the bone marrow, liver, and kidney^20^. Conversely, in this study, administration of FGF23 in wild-type mice resulted in a decrease in erythropoiesis^20^. These experimental evidence together with clinical research studies including ours suggest that FGF23 is a negative regulator of erythropoiesis. Unfortunately, a correlation between FGF23 and EPO could not be determined in our study because the EPO levels were not measured. Future studies are required to clarify the relationship between FGF23 and EPO.

Vitamin D deficiency has been suggested as a risk factor for anemia in patients with CKD. Previous studies from the Third National Health and Nutrition Examination Survey and Study to Evaluate Early Kidney Disease demonstrated that vitamin D deficiency was independently associated with anemia in patients with CKD^9,28^. Several studies using a burst-forming unit-erythroid assay have suggested a direct effect of vitamin D on proliferation of erythroid precursor cells obtained from patients with CKD, with a synergistic effect with EPO^29–31^. In addition, vitamin D deficiency is associated with secondary hyperparathyroidism, which can induce bone marrow fibrosis and suppress erythropoiesis in CKD^32^. Considering the fact that FGF23 decreases 1,25-dihydroxyvitamin D3 levels by inhibiting CYP27B1 (1-α-hydroxylase) and by stimulating CYP24A1 (24-hydroxylase)^10,11^, vitamin D deficiency may be a potential mechanistic link that can explain the relationship between FGF23 and anemia. In this study, however, the effect of FGF23 on the development of anemia was not altered after adjustment for 1,25-dihydroxyvitamin D3 levels. Moreover, there was no significant interaction between FGF23-related anemia and 1,25-dihydroxyvitamin D3 levels in subgroup analysis. These findings indirectly support the result from the aforementioned experimental study, in which abolishing vitamin D signaling from FGF23 null mice did not resolve the erythropoietic abnormalities^20^.

It is well known that iron deficiency is an important factor that can promote anemia in CKD. Interestingly, animal and human studies demonstrated that absolute and functional iron deficiency stimulates FGF23 production^33–36^. In line with these findings, our data showed that FGF23 was inversely correlated with iron profiles, including iron and TSAT, and positively correlated with hepcidin, which induces functional iron deficiency through iron sequestration and inhibition of iron absorption in the gastrointestinal tract^37^. Furthermore, subgroup analyses showed that a significant association between high FGF23 levels and the development of anemia was evident in patients with iron deficiency and high inflammatory status. It can be presumed that iron deficiency induces anemia either directly or indirectly through a negative impact of FGF23 on erythropoiesis.

Subgroup analyses showed that the use of RAS blockers can affect the relationship between FGF23 and the development of anemia. This association was evident in patients treated with RAS blockers (HR, 1.18; 95% CI, 1.07–1.29; P = 0.001), but not in patients without RAS blockers (HR, 1.04; 95% CI, 0.75–1.45; P = 0.81). Several experimental and clinical studies have suggested possible association between renin-angiotensin-aldosterone system and erythropoiesis^38–41^. Angiotensin II were demonstrated to be a physiologically important regulator of erythropoiesis, both as a growth factor of erythroid progenitors, and as an erythropoietin secretagogue to maintain increased erythropoietin^41^. In addition, serum aldosterone levels were demonstrated to play a role in the relationship between FGF23 and hemoglobin levels^21^. Moreover, RAS activation is reported to induce FGF23 resistance^42^. These findings together suggest that negative effect of FGF23 on erythropoiesis can be more evident in low renin-angiotensin-aldosterone status. Future studies are required to clarify the impact of RAS on FGF23-associated anemia.

Several shortcomings of this study should be discussed. First, because this is an observational study, it is possible that potential confounding factors were not entirely controlled. However, this study included a large number of patients and yielded consistent results in various multivariable Cox models after rigorous adjustment. Second, patients in our study had relatively higher eGFR than those in a previous study^21^; thus, the association between FGF23 and anemia needs to be verified in patients with advanced stages of CKD. Although there was no significant interaction between FGF23-related anemia and kidney function, subgroup analyses showed that association between FGF23 levels and incident anemia was particularly evident in patients with eGFR >30 ml·min^−1^·1.73 m^−2^. Furthermore, high FGF23 levels were also significantly associated with the future development of anemia in patients with low disease severity (e.g., with well-controlled BP, no diabetes, no obesity, and low comorbid conditions). Presumably, there are many other factors that can affect erythropoiesis in uremic conditions. These unseen factors seem to have overwhelmed the effect of FGF23 on anemia in patients with a high disease burden. Third, although iron deficiency modulates FGF23^33–36^, our study did not show that ferritin levels were correlated with FGF23 levels. However, it should be noted that ferritin is an acute-phase reactant and can be elevated in response to uremic inflammatory condition despite the presence of functional iron deficiency. This can explain a poor correlation between ferritin and FGF23 in CKD. Finally, we performed a single measurement of FGF23 concentrations at baseline and had no data for follow-up measurements. It would be interesting to see whether a change of FGF23 level is concordant to that of hemoglobin level. Further longitudinal studies are required to examine this relationship.

In conclusion, this study showed that serum FGF23 levels were inversely correlated with hemoglobin levels in patients with CKD and that patients with high FGF23 levels were more likely to have anemia. Furthermore, in patients without anemia at baseline, elevated FGF23 levels were associated with an increased risk of new development of anemia. Our findings suggest that FGF23 can be a useful predictor of anemia in patients with CKD. Further studies are required to clarify the mechanism for FGF23-associated anemia in these patients.

## Electronic supplementary material

Supplementary information

## References

[1] Astor BC, Muntner P, Levin A, Eustace JA, Coresh J (2002). Association of kidney function with anemia: the Third National Health and Nutrition Examination Survey (1988–1994). Arch Intern Med.

[2] Stauffer ME, Fan T (2014). Prevalence of anemia in chronic kidney disease in the United States. PLoS One.

[3] Levin, A. Anemia and left ventricular hypertrophy in chronic kidney disease populations: a review of the current state of knowledge. *Kidney Int Suppl*; 10.1046/j.1523-1755.61.s80.7.x. 35–38 (2002). 10.1046/j.1523-1755.61.s80.7.x 11982810

[4] Kovesdy CP, Trivedi BK, Kalantar-Zadeh K, Anderson JE (2006). Association of anemia with outcomes in men with moderate and severe chronic kidney disease. Kidney Int.

[5] Jurkovitz CT, Abramson JL, Vaccarino LV, Weintraub WS, McClellan WM (2003). Association of high serum creatinine and anemia increases the risk of coronary events: results from the prospective community-based atherosclerosis risk in communities (ARIC) study. J Am Soc Nephrol.

[6] Eschbach JW (1989). The anemia of chronic renal failure: pathophysiology and the effects of recombinant erythropoietin. Kidney Int.

[7] Weiss G, Goodnough LT (2005). Anemia of chronic disease. N Engl J Med.

[8] Nangaku M, Eckardt KU (2006). Pathogenesis of renal anemia. Semin Nephrol.

[9] Patel NM (2010). Vitamin D deficiency and anemia in early chronic kidney disease. Kidney Int.

[10] Martin A, David V, Quarles LD (2012). Regulation and function of the FGF23/klotho endocrine pathways. Physiol Rev.

[11] Gutierrez O (2005). Fibroblast growth factor-23 mitigates hyperphosphatemia but accentuates calcitriol deficiency in chronic kidney disease. J Am Soc Nephrol.

[12] Kovesdy CP, Quarles LD (2013). Fibroblast growth factor-23: what we know, what we don’t know, and what we need to know. Nephrol Dial Transplant.

[13] Fliser D (2007). Fibroblast growth factor 23 (FGF23) predicts progression of chronic kidney disease: the Mild to Moderate Kidney Disease (MMKD) Study. J Am Soc Nephrol.

[14] Isakova T (2011). Fibroblast growth factor 23 and risks of mortality and end-stage renal disease in patients with chronic kidney disease. Jama.

[15] Khan AM, Chirinos JA, Litt H, Yang W, Rosas SE (2012). FGF-23 and the progression of coronary arterial calcification in patients new to dialysis. Clin J Am Soc Nephrol.

[16] Gutierrez OM (2009). Fibroblast growth factor 23 and left ventricular hypertrophy in chronic kidney disease. Circulation.

[17] Kendrick J (2011). FGF-23 associates with death, cardiovascular events, and initiation of chronic dialysis. J Am Soc Nephrol.

[18] Scialla JJ (2014). Fibroblast growth factor-23 and cardiovascular events in CKD. J Am Soc Nephrol.

[19] Gutierrez OM (2008). Fibroblast growth factor 23 and mortality among patients undergoing hemodialysis. N Engl J Med.

[20] Coe LM (2014). FGF-23 is a negative regulator of prenatal and postnatal erythropoiesis. J Biol Chem.

[21] Tsai MH, Leu JG, Fang YW, Liou HH (2016). High Fibroblast Growth Factor 23 Levels Associated With Low Hemoglobin Levels in Patients With Chronic Kidney Disease Stages 3 and 4. Medicine (Baltimore).

[22] Eser B (2015). Fibroblast growth factor is associated to left ventricular mass index, anemia and low values of transferrin saturation. Nefrologia.

[23] Mehta, R. *et al*. Fibroblast Growth Factor 23 and Anemia in the Chronic Renal Insufficiency Cohort Study. *Clin J Am Soc Nephrol*; 10.2215/cjn.03950417. (2017). 10.2215/CJN.03950417 PMC5672973 28784656

[24] Oh KH (2014). KNOW-CKD (KoreaN cohort study for Outcome in patients With Chronic Kidney Disease): design and methods. BMC Nephrol.

[25] Levey AS (2006). Using standardized serum creatinine values in the modification of diet in renal disease study equation for estimating glomerular filtration rate. Ann Intern Med.

[26] Akalin N (2014). Prognostic importance of fibroblast growth factor-23 in dialysis patients. Int J Nephrol.

[27] Honda H (2017). High fibroblast growth factor 23 levels are associated with decreased ferritin levels and increased intravenous iron doses in hemodialysis patients. PLoS One.

[28] Kendrick J, Targher G, Smits G, Chonchol M (2009). 25-Hydroxyvitamin D deficiency and inflammation and their association with hemoglobin levels in chronic kidney disease. Am J Nephrol.

[29] Aucella F (2001). [Calcitriol increases burst forming unit-erythroid (BFU-E) *in vitro* proliferation in chronic uremia. Synergic effect with DNA recombinant erythropoietin (rHu-Epo)]. Minerva Urol Nefrol.

[30] Aucella F (2003). Calcitriol increases burst-forming unit-erythroid proliferation in chronic renal failure. A synergistic effect with r-HuEpo. Nephron Clin Pract.

[31] Alon DB (2002). Novel role of 1,25(OH)(2)D(3) in induction of erythroid progenitor cell proliferation. Exp Hematol.

[32] Brancaccio D, Cozzolino M, Gallieni M (2004). Hyperparathyroidism and anemia in uremic subjects: a combined therapeutic approach. J Am Soc Nephrol.

[33] David V (2016). Inflammation and functional iron deficiency regulate fibroblast growth factor 23 production. Kidney Int.

[34] Farrow EG (2011). Iron deficiency drives an autosomal dominant hypophosphatemic rickets (ADHR) phenotype in fibroblast growth factor-23 (Fgf23) knock-in mice. Proc Natl Acad Sci USA.

[35] Imel EA (2011). Iron modifies plasma FGF23 differently in autosomal dominant hypophosphatemic rickets and healthy humans. J Clin Endocrinol Metab.

[36] Wolf M, Koch TA, Bregman DB (2013). Effects of iron deficiency anemia and its treatment on fibroblast growth factor 23 and phosphate homeostasis in women. J Bone Miner Res.

[37] Babitt JL, Lin HY (2010). Molecular mechanisms of hepcidin regulation: implications for the anemia of CKD. Am J Kidney Dis.

[38] Kato H (2005). Enhanced erythropoiesis mediated by activation of the renin-angiotensin system via angiotensin II type 1a receptor. FASEB J.

[39] Cole J (2000). Lack of angiotensin II-facilitated erythropoiesis causes anemia in angiotensin-converting enzyme-deficient mice. J Clin Invest.

[40] Vlahakos DV (1999). Association between activation of the renin-angiotensin system and secondary erythrocytosis in patients with chronic obstructive pulmonary disease. Am J Med.

[41] Vlahakos DV, Marathias KP, Madias NE (2010). The role of the renin-angiotensin system in the regulation of erythropoiesis. Am J Kidney Dis.

[42] de Borst MH, Vervloet MG, ter Wee PM, Navis G (2011). Cross talk between the renin-angiotensin-aldosterone system and vitamin D-FGF-23-klotho in chronic kidney disease. J Am Soc Nephrol.

