# Supplementary material for: Circulating Fibroblast Growth Factor-23 Levels are Associated with an Increased Risk of Anemia Development in Patients with Nondialysis Chronic Kidney Disease
Source: Sci Rep. 2018 May 8;8:7294. doi: 10.1038/s41598-018-25439-z (PMC5940871; doi:10.1038/s41598-018-25439-z)
Supplement: Supplementary file 1 — Supplementary information [file 41598_2018_25439_MOESM1_ESM.docx]

**Circulating Fibroblast Growth Factor-23 Levels Are Associated with an Increased Risk of Anemia Development in Patients with Nondialysis Chronic Kidney Disease**

Ki Heon Nam^1^, Hyoungnae Kim^1^, Seong Yeong An^1^, Misol Lee^1^, Min-Uk Cha^1^, Jung Tak Park^1^, Tae-Hyun Yoo^1^, Kyu-Beck Lee^2^, Yeong-Hoon Kim^3^, Su-Ah Sung^4^, Joongyub Lee^5^, Shin-Wook Kang^1^, Kyu Hun Choi^1^, Curie Ahn^6^, and Seung Hyeok Han^1^

^1^Department of Internal Medicine, Yonsei University College of Medicine, Seoul, Korea; ^2^Department of Internal Medicine, Sungkyunkwan University School of Medicine, Kangbuk Samsung Hospital, Seoul, Korea; ^3^Department of Internal Medicine, Busan Paik Hospital, College of Medicine, Inje University, Busan, Korea; ^4^Department of Internal Medicine, Eulji General Hospital, Eulji School of Medicine, Seoul, Korea; ^5^Medical Research Collaborating Center, Seoul National University Hospital and Seoul National University College of Medicine, Seoul, Korea; ^6^Department of Internal Medicine, Seoul National University, Seoul, Korea

**Corresponding Author:**

Seung Hyeok Han, MD, PhD

Department of Internal Medicine, Institute of Kidney Disease Research, College of Medicine, Yonsei University

50-1 Yonsei-ro, Seodaemun-gu, Seoul 03722, Korea

Tel.: +82-2228-1984; Fax: +82-2393-6884

E-mail: hansh@yuhs.ac

**Supplementary table 1. Baseline characteristics of patients without baseline anemia**

| **Variables** | **FGF23 quartiles** | | | | **Total** | **p-value** |
| --- | --- | --- | --- | --- | --- | --- |
|  | **Quartile 1**  **(0.0-0.5)** | **Quartile 2**  **(0.6-13.1)** | **Quartile3**  **(13.2-28.6)** | **Quartile 4**  **(28.7-340.0)** |  |  |
| **Number** | 291 | 291 | 291 | 291 | 1,164 |  |
| **Age (years)** | 51.1±12.7 | 50.7±11.9 | 52.4±11.9 | 52.3±13.0 | 51.6±12.4 | 0.23 |
| **Sex (Male, %)** | 195 (67.0) | 172 (59.1) | 204 (70.1) | 191 (65.6) | 762 (65.5) | 0.04 |
| **Smoking (n, %)** | 143 (49.1) | 124 (42.6) | 154 (53.1) | 144 (49.5) | 565 (48.6) | 0.08 |
| **DM (n, %)** | 56 (19.2) | 52 (20.6) | 66 (26.1) | 79 (27.1) | 253 (21.7) | 0.03 |
| **HTN (n, %)** | 269 (92.4) | 278 (95.5) | 278 (95.5) | 281 (96.6) | 1106 (95.0) | 0.12 |
| **BMI (kg/m^2^)** | 24.8±3.3 | 24.7±3.3 | 25.0±3.6 | 24.8±3.6 | 24.8±3.5 | 0.64 |
| **SBP (mmHg)** | 127.3±14.8 | 126.7±14.2 | 126.9±14.9 | 128.9±15.4 | 127.5±14.8 | 0.28 |
| **DBP (mmHg)** | 77.5±11.4 | 77.2±10.2 | 77.5±10.9 | 78.5±10.7 | 77.7±10.8 | 0.49 |
| **MAP (mmHg)** | 94.1±11.5 | 93.7±10.4 | 94.0±11.3 | 95.3±11.1 | 94.3±11.1 | 0.32 |
| **Charlson comorbidity index** | 1.4±1.4 | 1.5±1.5 | 2.0±1.6 | 2.0±1.5 | 1.7±1.5 | <0.001 |
| **Creatinine (mg/dl)** | 1.2±0.5 | 1.2±0.5 | 1.4±0.6 | 1.5±0.8 | 1.3±0.6 | <0.001 |
| **eGFR (ml/min/1.73m^2^)** | 69.1±28.0 | 68.0±31.3 | 57.2±25.3 | 58.7±30.9 | 63.3±29.5 | <0.001 |
| **CKD stage** |  |  |  |  |  |  |
| **1 (n, %)** | 91 (31.3) | 89 (30.6) | 51 (17.5) | 68 (23.4) | 299 (25.7) | <0.001 |
| **2 (n, %)** | 92 (31.6) | 78 (26.8) | 77 (26.5) | 68 (23.4) | 315 (27.1) | 0.16 |
| **3 (n, %)** | 91 (31.3) | 112 (38.5) | 135 (46.4) | 106 (36.4) | 444 (38.1) | 0.002 |
| **4 (n, %)** | 17 (5.8) | 11 (3.8) | 27 (9.3) | 44 (15.1) | 99 (8.5) | <0.001 |
| **5 (n, %)** | 0 (0.0) | 1 (0.3) | 1 (0.3) | 5 (1.7) | 7 (0.6) | 0.04 |
| **WBC (x10^3^/mm^3^)** | 6.5±2.0 | 6.6±1.8 | 6.8±1.8 | 6.8±2.0 | 6.7±1.9 | 0.06 |
| **Hemoglobin (g/dl)** | 14.3±1.2 | 14.3±1.4 | 14.3±1.3 | 14.2±1.4 | 14.3±1.3 | 0.81 |
| **Platelet (x10^3^/mm^3^)** | 228.4±61.1 | 226.9±49.6 | 231.5±56.1 | 233.5±59.0 | 230.1±56.6 | 0.49 |
| **Iron (µg/dl)** | 102.2±34.9 | 104.4±37.4 | 107.3±37.1 | 97.4±33.6 | 102.8±35.9 | 0.008 |
| **Ferritin (ng/ml)*** | 109.4  (62.8-177.8) | 86.8  (52.0-165.6) | 96.4  (53.7-175.7) | 97.5  (47.3-170.8) | 98.0  (54.5-172.2) | 0.25 |
| **Transferrin saturation (%)** | 33.1±11.2 | 34.4±13.1 | 34.9±12.5 | 32.0±11.6 | 33.6±12.1 | 0.02 |
| **Hepcidin (ng/ml)*** | 10.6  (6.0-19.6) | 10.1  (5.7-18.0) | 12.3  (6.5-19.0) | 12.1  (6.4-21.2) | 11.3  (6.1-19.2) | 0.19 |
| **Total cholesterol (mg/dl)** | 179.7±37.1 | 178.7±36.8 | 178.2±37.6 | 176.3±40.1 | 178.2±37.9 | 0.75 |
| **Albumin (g/dl)** | 4.3±0.3 | 4.3±0.3 | 4.3±0.4 | 4.3±0.4 | 4.3±0.3 | 0.07 |
| **Calcium (mg/dl)** | 9.3±0.4 | 9.3±0.4 | 9.3±0.4 | 9.3±0.5 | 9.3±0.4 | 0.85 |
| **Phosphate (mg/dl)** | 3.4±0.5 | 3.5±0.3 | 3.5±0.5 | 3.5±0.6 | 3.5±0.5 | 0.61 |
| **iPTH (pg/ml)*** | 38.6  (27.0-57.6) | 42.3  (28.9-59.6) | 29.9  (44.1-64.4) | 49.8  (32.8-75.1) | 43.5  (29.0-63.6) | <0.001 |
| **1,25(OH)2 vitamin D (pg/ml)** | 36.2±18.2 | 31.1±14.1 | 30.1±13.0 | 29.7±14.1 | 31.7±15.2 | <0.001 |
| **FGF23 (RU/ml)*** | 0.01  (0.0-0.1) | 4.8  (1.8-8.3) | 20.3  (18.1-23.6) | 32.5  (40.0-55.2) | 13.1  (0.5-28.6) | <0.001 |
| **CRP (mg/dl)*** | 0.7 (0.2-1.6) | 0.5 (0.2-1.3) | 0.6 (0.3-1.4) | 0.6 (0.2-1.8) | 0.6 (0.2-1.5) | 0.60 |
| **Proteinuria (g/24h)*** | 0.2  (0.1-0.8) | 0.4  (0.1-0.8) | 0.5  (0.1-1.5) | 0.5  (0.1-1.3) | 0.4  (0.1-1.0) | <0.001 |
| **Treatment** |  |  |  |  |  |  |
| **RAS blockers (n, %)** | 250 (85.9) | 258 (88.7) | 251 (86.3) | 252 (86.6) | 1011 (86.9) | 0.76 |
| **Statin (n, %)** | 150 (51.9) | 152 (52.2) | 144 (49.5) | 148 (51.0) | 594 (51.2) | 0.91 |
| **Iron replacement (n, %)** | 5 (1.7) | 14 (4.8) | 9 (3.1) | 22 (7.6) | 50 (4.3) | 0.004 |
| **ESA therapy (n, %)** | 2 (0.7) | 2 (0.7) | 4 (1.4) | 8 (2.7) | 16 (1.4) | 0.11 |

All data are expressed as mean ± SD or *median (and interquartile range)

Abbreviations: FGF23, C-terminal fibroblast growth factor 23; DM, diabetes mellitus; HTN, hypertension; BMI, body mass index; SBP, systolic blood pressure; DBP, diastolic blood pressure; MAP, mean arterial pressure; eGFR, estimated glomerular filtration rate; CKD, chronic kidney disease; WBC, white blood cell; iPTH, intact parathyroid hormone; CRP, C-reactive protein; RAS, renin-angiotensin system; ESA, erythropoiesis-stimulating agent
